# Effect of Performance Speed on Trunk Movement Control During the Curl-Up Exercise

**DOI:** 10.1515/hukin-2015-0031

**Published:** 2015-07-10

**Authors:** David Barbado, Jose Luis L. Elvira, Francisco J. Moreno, Francisco J. Vera-Garcia

**Affiliations:** 1 Sports Research Centre, Miguel Hernandez University of Elche, Elche (Alicante), Spain.

**Keywords:** conditioning exercise, abdominal musculature, velocity, motor control, spine biomechanics

## Abstract

Trunk exercise speed has significant effects on neuro-mechanical demands; however, the influence of a variety of exercise speeds on motor control of the trunk displacement remains unknown. The aim of this study was to assess the effect of performance speed on trunk motion control during the curl-up exercise by analyzing the kinematic variance about the sagittal trajectory. Seventeen subjects volunteered to perform curl-ups at different cadences controlled by a metronome. Standard deviation (SD) and range (RG) of shoulder girdle medial-lateral displacement (SG_ML_) and detrended fluctuation analysis (DFA) of SG_ML_ were calculated to examine linear variability and long range autocorrelation of medial-lateral upper trunk displacements, respectively. In addition, SD, RG and DFA of centre of pressure medial-lateral displacement (COP_ML_) were performed to analyze the behavior of the motor system while controlling trunk displacement. Although SD and RG of COP_ML_ increased as speed increased, the curl-up cadence did not have significant effects on SD and RG of SG_ML_. These results suggest that although high speed curl-ups challenged participants’ ability to carry out medial-lateral adjustments, an increase of performance speed did not modify the linear variability about the sagittal trajectory. Regarding DFA, the scaling exponent α of SG_ML_ and COP_ML_ was higher for the fastest movements, mainly in long term fluctuations. Therefore, to maintain the target trajectory, participants used different strategies depending on performance speed. This is to say, there were less trajectory changes when participants performed the fastest exercises.

## Introduction

Trunk flexion exercises are broadly used for abdominal muscle conditioning in sport, fitness, and rehabilitation. Research has primarily focused on the effect of hip flexion, supported segments, arm and hand position, knee and hip position, movement of the upper body vs. lower body and the use of equipment, on trunk muscle response (Monfort-Pañego et al., 2009) and spine loading (Axler and McGill, 1997; Kavcic et al., 2004). However, although performance speed is an important variable to prescribe trunk exercise programs (Bird et al., 2005; Vera-Garcia et al., 2008), scientific evidence of the effect of exercise speed on trunk neuromuscular control and mechanics is lacking.

Previous electromyographic studies comparing different trunk flexion speeds showed that the highest exercise speeds required the highest activation levels of flexor and extensor trunk muscles (Vera-Garcia et al., 2008). An increase in muscular coactivation with trunk exercise speed seems to be related to the development of muscular forces involved in at least two conflicting functions, this is, producing rapid-plyometric trunk motions and ensuring spine stability (McGill, 2006).

A recent study on the effect of movement speed on the kinematics and kinetics of trunk and hip strengthening exercises (curl-up, sit-up and double leg raising/lowering) found variations in exercise technique resulting from speed increases (Elvira et al., 2014). In relation to the curl-up or crunch exercise, as performance speed increased, the trunk, hip and knee sagittal range of motion and cephalo-caudal centre of pressure (COP) displacement increased. According to Elvira et al. (2014), these results could be due to increased angular momentum with speed and may impact training results.

Moreover, several biomechanical studies on standing tasks (mainly lifting tasks) have shown the impact of sagittal trunk motion speed on muscle forces, spinal loads, trunk movement control and the risk of low-back injuries (Davis and Marras, 2000; Granata and England, 2006). In this way, Granata and England (2006) performed a study on the effect of trunk flexion-extension speed on trunk dynamic stability in standing, in which they found that the motor system’s ability to maintain a desired trunk trajectory decreased as trunk movement was executed at a faster pace.

Based on the results of the aforementioned studies, trunk exercise speed is a variable that requires close monitoring and control while training, as it has a significant effect on neuromuscular (Vera-Garcia et al., 2008) and mechanical demands (Elvira et al., 2014); however, the influence of a variety of exercise speeds on trunk movement control has not been fully understood and needs further research. In this sense, although increasing curl-up exercise speed increases trunk muscle activation (Vera-Garcia et al., 2008), which can be suitable for trunk muscle strengthening, it can also hinder trunk control and as a result increase injury risk. Consequently, finding the maximum trunk exercise speed in which trunk motion control is not impaired could help to improve the efficiency of the abdominal training programs without increasing injury risk.

Therefore, the purpose of this study was to assess the effect of curl-up exercise speed on the control of trunk motion through the analysis of linear and non-linear variability of medial-lateral trunk displacements. Participants were asked to perform curl-ups at four different cadences while trying to adjust their movement to the sagittal plane without visual or auditory feedback. Specifically, standard deviation (SD) and range (RG) of the shoulder girdle medial-lateral displacement (SG_ML_) were measured to assess the participants’ ability to constrain the upper trunk motion to the sagittal plane. In addition, detrended fluctuation analysis (DFA), previously used to assess motion and postural adjustments (Amoud et al., 2007; Jordan et al., 2007; Wang and Yang, 2012), was carried out to evaluate the participants’ ability to perform SG_ML_ corrections while executing the curl-ups. Finally, the SD, RG and DFA of COP medial lateral displacement (COP_ML_) were also measured in order to enable a discussion around the objective of a better understanding of the behavior/strategies of the motor system to control the upper trunk trajectory during these exercises. Overall, we were motivated to obtain a deeper insight into the control of the trunk motion to ultimately establish which trunk curl-up speed maximizes muscle activation while assuring trunk dynamic stability. This may provide useful information to assist coaches and practitioners in prescribing trunk exercise programs.

## Material and Methods

### Participants

Seventeen asymptomatic volunteers (13 females and 4 males), recruited from a university population, took part in this study (age: 23.58 ± 4.43 years; body height: 166.27 ± 6.47 cm; body mass: 61.00 ± 8.40 kg). Subjects with known medical problems, histories of spinal, shoulder or hip surgery or episodes of back pain requiring treatment twelve months before this study were excluded. All participants were recreationally physically active and performed trunk flexion exercises with a frequency of 1–3 days per week.

Written informed consent was obtained from each participant prior to testing. The experimental procedures used in this study were in accordance with the Declaration of Helsinki and were approved by the Committee for Research Ethics at the Miguel Hernandez University of Elche (Spain).

### Experimental procedures

The participants were asked to perform curl-ups at four different cadences controlled by a metronome: 1 repetition/4 s (C4), 1 repetition/2 s (C2), 1 repetition/1.5 s (C1.5), and 1 repetition/1 s (C1). They performed seven consecutive repetitions in each cadence, with a 2 min rest period between trials to avoid muscle fatigue. Cadences were counterbalanced between subjects. The first and last repetitions of each trial were discarded from the analysis.

**Figure 1:**
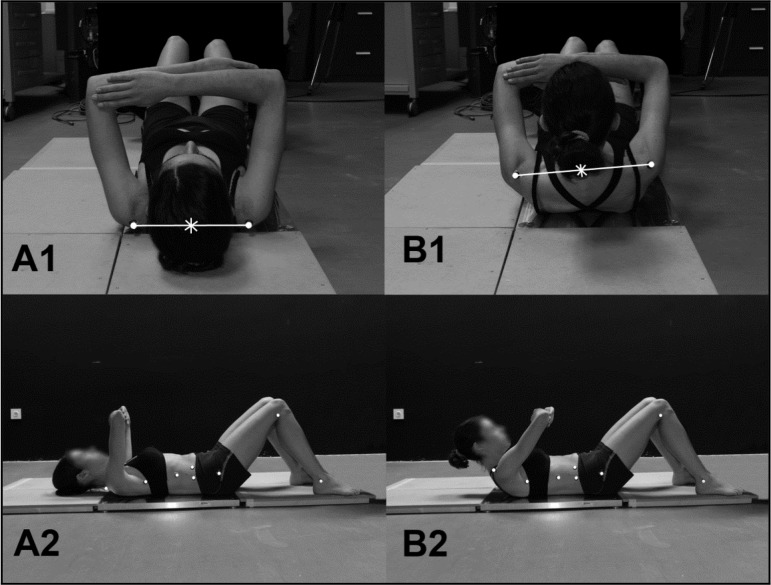
The posterior (1) and lateral (2) view of the initial position (A) and trunk curled position (B) of a subject while performing the curl-up exercise. The midpoint of the reflective markers represents the movement of the shoulder girdle

Curl-ups started from a lying supine position with the trunk on a force plate (600×370 mm, Dinascan 600M, IBV, Valencia, Spain), knees flexed at 90º and feet resting on the floor, outside the force plate (Figure 1). Shoulders and elbows were flexed at 90º with the hands placed on the opposite elbow and the forehands maintained in front of the chest. Curl-ups consisted of a head, arms and upper trunk lift to the point where the scapula was lifted from the force plate (Figure 1), then returning to the starting position. Participants were encouraged to avoid non-sagittal movements, but they did not receive feedback from the researchers during the performance. Before data collection, each participant practiced the different cadences until they learned the correct rhythm of the movement.

Before starting each trial, the region from the participant’s shoulders to the pelvis was resting on the force plate and care was taken to align the sagittal plane of the trunk motion to the longer axis of the force plate. In this initial position, the participants’ mean mass rested on the force plate was 44.82 ± 5.85 kg, which represented 75.64 ± 1.70% of their mean total mass. Reliability of the participants’ initial position between trials was good for both absolute (ICC = 0.99; SEM = 1.27%) and relative body mass measure (ICC = 0.79; SEM = 1.39%).

Ground reaction forces were recorded at 100 Hz during the exercise execution, and the COP_ML_ was calculated. Following data collection, the data were filtered at 20 Hz, with a low-pass fourth-order Butterworth filter.

Simultaneously, a 3D photogrammetric analysis was performed. Three digital cameras (Canon XM1, Sony DCRTRV33 and Sony SSCDC338) recording at 50 Hz were placed at 0º, 45º and 90º from the sagittal plane. The reference frame used was a prism of 2x1x1 m aligned with the force plate reference system. The markers were automatically digitized and reconstructed with the software Kwon 3D (Visol Inc., Korea). The movement of the shoulder girdle was depicted by the displacement of the midpoint of the reflective markers placed on the shoulders (Figure 1) (Kwon, 1996). Following data collection, the kinematic data were filtered at 10 Hz, with a low-pass second-order Butterworth filter.

### Data analyses

As explained before, SD and RG of the SG_ML_ and DFA of the SG_ML_ were calculated to analyze linear and non-linear kinematic variability of medial-lateral trunk displacements, respectively. In addition, SD, RG and DFA of the COP_ML_ were performed to analyze the behavior/strategies of the motor system while trying to control the upper trunk sagittal trajectory.

The DFA method had been previously used both to evaluate the effect of speed on gait cycle stability during treadmill running by analyzing the motor system’s competence to perform gait adjustments (Jordan et al., 2007), and to assess postural adjustments while upright standing in elderly subjects (Amoud et al., 2007; Wang and Yang, 2012). DFA investigates long range correlation contained within the time series by a parameter referred to as the scaling index *α* (Bashana et al., 2008; Shao et al., 2012). It was specially designed for the analysis of biological time series for two reasons: 1) DFA avoids the problem of biological signal boundaries, because the time series is first integrated (Delignières et al., 2006); and 2) DFA can be used in short data series (Delignières et al., 2006), which was important in this study as we avoided the problem of fatigue in long trials.

To calculate the scaling index *α*, DFA includes a series of operations: firstly, the analyzed series *X(t)* is integrated, by computing for each *t* the accumulated departure from the mean of the whole series:

$$X(k)=\sum_{i=1}^{k}[x(i)-\bar{x}]$$

Secondly, the integrated series *X(k)* is then divided into non-overlapping intervals of length *n*. In each interval, the least squares regression line (representing the local trend within the interval) is fitted to the data. The series *X(k)* is then locally detrended by subtracting the theoretical values *X_n_(k)* given by the regression. Finally, for each interval length *n*, the characteristic size of the fluctuation for this integrated and detrended series is given by:

$$F(n)=\sqrt{\frac{1}{N}\sum_{k=1}^{N}{\left[X(k)-X_{n}(k)\right]}^{2}}$$

This computation is repeated over different segment lengths to yield the index *F(n)* as a function of segment length *n*. Typically *F(n)* increases with segment length. A linear relationship on a double log graph indicates a degree of correlation characterized by the scaling exponent *α* (the slope of the regression line relating log *F(n)* to log *n*). Different values of *α* indicate the following: *α* > 0.5 implies persistence (i.e., the trajectory tends to continue in its current direction); *α* < 0.5 implies anti-persistence (i.e., the trajectory tends to return to where it came from); *α* > 1 implies the signal is not stationary (Eke et al., 2002); α = 0.5 implies uncorrelated signal.

Studies that examined the temporal structure of the centre of pressure have related a less dependent structure (less persistent autocorrelation) with an increase of flexibility of the system to carry out motion adjustments (Amoud et al., 2007; Jordan et al., 2007; Wang and Yang, 2012).

In our study, two window ranges were calculated in order to differentiate long-term and short-term fluctuations. In order to maximize the long range correlations and to reduce the error in the estimation of *α*, a long term correlation was characterized by the slope *α*_2_ obtained from the range 4 ≤ *n* ≤ *N*/10 (Chen et al., 2002). In our study, this range corresponds with the time required to complete half a cycle. A short-term correlation was characterized by the slope *α*_1_ obtained from the range 4 ≤ *n* ≤ *N*/25.

The number of data per cycle can influence the estimation of long range correlations (Deffeyes et al., 2009). Therefore, the COP_ML_ data were resampled to obtain 500 data samples per 5 cycles. We selected 500 samples interpolation because it was the minimum amount of data needed to avoid aliasing in the slowest cadence.

### Statistical analyses

Data normality was examined using the Kolmogorov-Smirnov statistic with a Lilliefors correction. One-way repeated-measures ANOVAs were performed in order to investigate the effects of increasing curl-up speed (cadences: C4, C2, C1.5, C1) on SD, RG and DFA of the SG_ML_ and COP_ML_. Post hoc analysis with Bonferroni adjustment was used for multiple comparisons. Mass and height were used as covariates but they showed no significant effects on any ANOVA. Partial eta squared ( $\eta_{p}^{2}$) was calculated as a measure of effect size. Values of effect size ≥0.64 were considered strong, from 0.25–0.64 they were considered moderate and ≤0.04 were considered small (Ferguson, 2008). All analyses were performed using the SPSS package version 20.0 (IBM SPSS Inc., Chicago, IL, USA) with a significance level set at p < 0.05.

## Results

**Figure 2:**
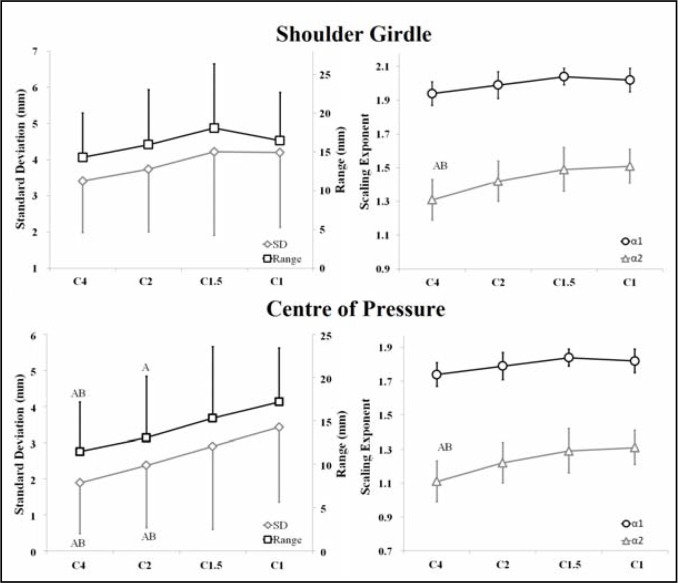
Effects of the curl-up cadence (C4: 1 repetition/4 s; C2: 1 repetition/2 s; C1.5: 1 repetition/1.5 s; C1: 1 repetition/1 s) on standard deviation, range, scaling exponents α_1_ and α_2_ (short and long term) of the medial-lateral displacement of the centre of pressure and shoulder girdle. ANOVA for repeated measures: ^A^Significantly different from C1 with p < 0.05; ^B^Significantly different from C1.5 with p < 0.05. Bonferroni adjustment was used for multiple comparisons.

As it can be seen in Figure 2, although the SD and RG of SG_ML_ did not show significant differences between curl-up cadences (SD: F_3,48_ = 1.940, p = 0.136, $\eta_{p}^{2}$ = 0.101; RG: F_3,48_= 2.194, p = 0.101, $\eta_{p}^{2}$ = 0.121), the scaling exponents *α_1_* and *α_2_* of SG_ML_ increased as speed increased (*α_1_*: F_3,48_ = 2.761, p = 0.052, $\eta_{p}^{2}$ = 0.147; *α_2_*: F_3,48_ = 7.825, p = 0.001, $\eta_{p}^{2}$ = 0.313). In addition, the SD, RG and scaling exponents *α_1_* and *α_2_* of COP_ML_ were significantly higher for the faster curl-up cadences (SD: F_3,48_ = 15.378, p < 0.001, $\eta_{p}^{2}$ = 0.475; RG: F_3,48_ = 15.378, p < 0.001, $\eta_{p}^{2}$ = 0.414; *α_1_*: F_3,48_ = 11.491, p < 0.001 $\eta_{p}^{2}$ = 0.403; *α_2_*: F_3,48_ = 17.073, p < 0.001, $\eta_{p}^{2}$ = 0.501). In all cadences, scaling exponent *α_1_* of SG_ML_ and COP_ML_ was higher than *α_2_* (Figure 2).

## Discussion

This research assesses the effect of curl-up exercise speed on the participants’ ability to constrain the trunk flexion motion to the sagittal plane by analyzing the kinematic linear and nonlinear variance about the sagittal trajectory. Our main finding was that linear variability of SG_ML_ did not change significantly as speed increased. However, the linear variability of COP_ML_ increased and the SG_ML_ and COP_ML_ became more persistent when trunk motion speed increased. These results indicate that although high speed curl-ups challenged participants’ ability to carry out medial-lateral adjustments (as shown by the increase of COP_ML_ variability); they were able to constrain their upper trunk motion to the sagittal plane without significant changes between cadences.

The non-significant effect of curl-up speed on linear variability of SG_ML_ in our research does not agree with Granata and England’s results (2006), as they observed a reduction of the motor system’s ability to maintain a desired trunk trajectory (i.e., a reduction of dynamic trunk stability) as speed increased during repetitive flexion-extension movements in standing. In addition, traditional studies on speed-accuracy trade-off in aimed movements found higher errors when the movement was performed at a faster pace (Etnyre, 1998; Fitts, 1954). Possibly, the differences between studies could be due to our participants’ expertise in performing curl up exercises, as they performed curl-ups with a frequency of 1–3 days per week. In these sense, Beilock et al. (2008) and García et al. (2013) observed that the level of expertise/training reduced the loss of accuracy in striking and throwing tasks when performance speed increased. The differences between our and Granata and England’s results could also be explained by the differences in task constraints among studies. While in Granata and England’s research (2006) participants executed repetitive trunk and hip flexion-extension movements in standing, touching reference targets, in our study participants performed upper trunk flexion-extension movements in supine with the lumbar region supported on the ground and without visual feedback. Therefore, task experience of our participants and the little difficulty of curl-up performance (small range of motion and lower trunk support) may explain the non-significant effect of performance speed on medial-lateral sway. Nevertheless, the interaction between trunk motion control, task experience, task constraints and performance speed must be explored in future studies.

As Figure 2 shows, the linear variability of COP_ML_ significantly increased as curl-up exercise speed increased. Taking into account that COP fluctuations reflect the neuromuscular system’s response to control the body motion (Winter, 1990), our results suggest that compared to the slowest curl-up exercises, participants performed a greater neuromuscular effort to control trunk motion during the fastest curl-up exercises. Similarly, Elvira et al. (2014) found higher cephalo-caudal COP displacement and greater difficulty to slow down the trunk flexion motion as curl-up speed increased, which appeared to be a side effect of increasing the angular momentum with speed. These and our results are consistent with those of Vera-Garcia et al. (2008), which compared the trunk muscle response during the execution of curl-ups at different cadences (C4, C2, C1.5 and C1) in a sample with similar characteristics (age, anthropometry, weekly psychical activity, etc.) to those of our participants. They found that the highest curl-up speeds required the highest levels of trunk muscle coactivation. Higher demands on the motor system modulated by performance speed increases may be desirable for specific stages in a training program. However, due to the effect of performance speed on the spinal loads and intradiscal pressure (Axler and McGill, 1997), fast curl-up exercises should be used with caution in people with motor control deficits or low-back disorders, as well as in novice, untrained or unfit individuals.

In relation to the DFA results, we observed an increment of the persistent autocorrelation as speed increased in curl-up exercises. Indexes α_1_ and α_2_ showed that SG_ML_ and COP_ML_ were largely determined by previous medial-lateral displacements in the highest speed exercises. These findings suggest that performance speed modified the way participants adjusted their upper trunk motion to the sagittal plane. Possibly, the exercise speed increases constrained the motor system’s ability to perform fast changes of the upper trunk motion, leading to a nearly straight upper trunk trajectory, as reported previously in fast cyclic aimed movements (Djioua and Plamondon, 2010). Some factors may explain the limitations of the motor system to modify the upper trunk trajectory during rapid movements. First, fast movements reduce the available time for neuromuscular corrections (Ogata, 2002). In this sense, compared to α_2_ values, the higher α_1_ values of SG_ML_ and COP_ML_ found in all cadences in this study point out the difficulty to perform medial-lateral adjustments when there is little time available. Second, trunk momentum increases as exercise speed increases (Elvira et al., 2014), requiring a higher neuromuscular effort to change trunk trajectory if necessary (Granata and England, 2006). In addition, trunk muscle activity and co-contraction increase during fast plyometric movements (Dolan and Adams, 1993; McGill, 1995; Vera-Garcia et al., 2008). In this sense, high levels of co-contraction during the fastest curl-ups could increase trunk stiffness (Cholewicki and McGill, 1996; Vera-Garcia et al., 2006) and limit the ability to perform fine changes of medial lateral motion during fast movements, as the regulation of muscle forces when muscle activity is high requires the recruitment of large motor units (Granata and England, 2006).

In conclusion, the results of this study suggest that although high speed curl-ups challenged the participants’ ability to carry out medial-lateral adjustments, the increase of performance speed did not modify the linear variability about the sagittal trajectory. To maintain the target trajectory, participants used different strategies depending on performance speed. As shown by the increase of the scaling exponent α_1_ and α_2_ of SG_ML_ and COP_ML_, there were less trajectory changes when participants performed the fastest exercises.

Based on these and previous EMG results (Vera-Garcia et al., 2008), the fastest cadence in our study (1 repetition/1 s) could be used in young physically active individuals to produce high levels of trunk muscle activation without impairing trunk motion control, therefore allowing practitioners to improve the efficiency of the abdominal training programs targeted to this population. However, fast curl-up cadences may hinder trunk control in other populations and therefore not benefit them in the same way. In this sense, novice, unfit or older individuals may need slower curl-up cadences to maximize the efficiency of the abdominal training programs without increasing injury risk due to the lack of trunk movement control and/or high spinal loading. Future studies should assess the effects of trunk exercise speed in these and other populations, such as low back pain patients or high level athletes. In addition, further studies should simultaneously assess kinematic, kinetic and electromyographic measures in order to obtain more comprehensive knowledge about to what extent abdominal exercise speed modulates the relationship between trunk neuromuscular response and motion control.
